# Selection of Appropriate Reference Genes for RT-qPCR Analysis in a Streptozotocin-Induced Alzheimer’s Disease Model of Cynomolgus Monkeys (*Macaca fascicularis*)

**DOI:** 10.1371/journal.pone.0056034

**Published:** 2013-02-14

**Authors:** Sang-Je Park, Young-Hyun Kim, Youngjeon Lee, Kyoung-Min Kim, Heui-Soo Kim, Sang-Rae Lee, Sun-Uk Kim, Sang-Hyun Kim, Ji-Su Kim, Kang-Jin Jeong, Kyoung-Min Lee, Jae-Won Huh, Kyu-Tae Chang

**Affiliations:** 1 National Primate Research Center, Korea Research Institute of Bioscience and Biotechnology, Ochang, Chungbuk, Republic of Korea; 2 Department of Biological Sciences, College of Natural Sciences, Pusan National University, Busan, Republic of Korea; 3 University of Science & Technology, National Primate Research Center, Korea Research Institute of Bioscience and Biotechnology, Chungbuk, Republic of Korea; 4 Department of Rehabilitation Science in Interdisciplinary PhD Program, Graduate School of Inje University, Gimhae, Gyeongnam, Republic of Korea; 5 Department of Neurology, Seoul National University Hospital, Seoul, Republic of Korea; Oregon Health & Science University, United States of America

## Abstract

Reverse transcription quantitative real-time polymerase chain reaction (RT-qPCR) has been widely used to quantify relative gene expression because of the specificity, sensitivity, and accuracy of this technique. In order to obtain reliable gene expression data from RT-qPCR experiments, it is important to utilize optimal reference genes for the normalization of target gene expression under varied experimental conditions. Previously, we developed and validated a novel icv-STZ cynomolgus monkey model for Alzheimer’s disease (AD) research. However, in order to enhance the reliability of this disease model, appropriate reference genes must be selected to allow meaningful analysis of the gene expression levels in the icv-STZ cynomolgus monkey brain. In this study, we assessed the expression stability of 9 candidate reference genes in 2 matched-pair brain samples (5 regions) of control cynomolgus monkeys and those who had received intracerebroventricular injection of streptozotocin (icv-STZ). Three well-known analytical programs geNorm, NormFinder, and BestKeeper were used to choose the suitable reference genes from the total sample group, control group, and icv-STZ group. Combination analysis of the 3 different programs clearly indicated that the ideal reference genes are *RPS19* and *YWHAZ* in the total sample group, *GAPDH* and *RPS19* in the control group, and *ACTB* and *GAPDH* in the icv-STZ group. Additionally, we validated the normalization accuracy of the most appropriate reference genes (*RPS19* and *YWHAZ*) by comparison with the least stable gene (*TBP*) using quantification of the *APP* and *MAPT* genes in the total sample group. To the best of our knowledge, this research is the first study to identify and validate the appropriate reference genes in cynomolgus monkey brains. These findings provide useful information for future studies involving the expression of target genes in the cynomolgus monkey.

## Introduction

Reverse transcription quantitative real-time polymerase chain reaction (RT-qPCR) is a widely used experimental method for the detection and evaluation of mRNA levels because of the specificity, accuracy, sensitivity, and cost-effectiveness. Despite these advantages, a number of parameters such as differing sample amounts, RNA quality, purity, enzymatic efficiency in reverse transcription, and PCR efficiency can lead to inaccurate quantification of gene expression data by using RT-qPCR experiments [1]. To overcome this problem, normalization strategies are commonly used with constitutively expressed gene, termed the reference gene or the internal control gene [2]. Traditional reference genes such as *glyceraldehyde-3-phosphate dehydrogenase* (*GAPDH*) and *β-actin* (*ACTB*) are frequently used to normalize the target gene expression levels. However, these genes have been shown to have variable expression patterns across tissue types and experimental conditions [1], [3]. Therefore, selection of suitable reference genes is important to avoid incorrect results obtained from differential expression patterns in specific tissue types and experimental conditions [4].

Previous studies have shown that rodents injected intracerebroventricular streptozotocin (icv-STZ) show pathological features similar to those of Alzheimer’s disease (AD), such as neuronal loss, accumulation of Aβ, hyperphosphorylation of tau, and impairment of spatial learning. In particular, a chronic decrease in cerebral glucose uptake and production has been observed in icv-STZ rats [5], [6]. Abnormalities of brain glucose metabolism are major features of early stages of AD [7], and therefore, the icv-STZ rat is a useful animal model for the investigation of AD. However, rodent models are not ideal for the investigation of spatial distribution and regional differences in pathogenetic vulnerability because the small rodent brains do not allow detailed spatial mapping. To better understand spatial- and regional-specific differences in the pathological features of AD, we established a primate model of AD using icv-STZ of cynomolgus monkeys [8]. Interestingly, 18F-deoxyglucose uptake monitoring by high-resolution micro-PET of this primate model showed hypometabolism of glucose. However, this study has not been performed molecular characterization of AD-related genes in the detailed region of icv-STZ monkey brain. Therefore, studies are needed to characterize the genes showing altered expression in the icv-STZ primate model brain. In order to further investigate this model, it is important to determine the appropriate reference genes before completing experiments on target genes such as those involved in the insulin signaling pathway and AD-related genes in the brain tissues of normal and icv-STZ primate models. To our knowledge, studies designed to select suitable reference genes have not been performed in cynomolgus monkeys. In order to provide a more suitable model organism for studies of neurodegenerative diseases such as AD and Parkinson’s disease (PD), appropriate reference genes must be identified for RT-qPCR experiments in a range of regions in normal and icv-STZ brains.

The aim of this study was to select and evaluate the stability of 9 candidate reference genes in order to identify reliable genes that may be used for normalization when studying AD-related gene expression. To this end, samples from different regions of control and icv-STZ cynomolgus monkey brains were used. The stability *ACTB, β-2-microglobulin* (*B2M*), *GAPDH, hypoxanthine phosphoribosyltransferase 1* (*HPRT1*), *ribosomal protein S5* (*RPS5*), *RPS19*, *succinate dehydrogenase complex subunit A* (*SDHA*), *TATA box binding protein* (*TPB*), and *tyrosine 3-monooxygenase/tryptophan 5-monooxygenase activation protein ζ polypeptide* (*YWHAZ*) were analyzed using the geNorm [1], NormFinder [9], and BestKeeper [10] software programs. In addition, we validated the reference genes identified in our study by comparison with the mRNA levels of *amyloid beta (A4) precursor protein* (*APP*) and *microtubule-associated protein tau* (*MAPT*) in the control and icv-STZ brains.

## Results

### Expression Stability of Candidate Reference Genes

We analyzed the 9 candidate genes to select the most stable and suitable reference genes using 3 software programs (geNorm, NormFinder, and BestKeeper). These are freely available and generally accepted tools.

#### a) GeNorm analysis

**Figure 1:**
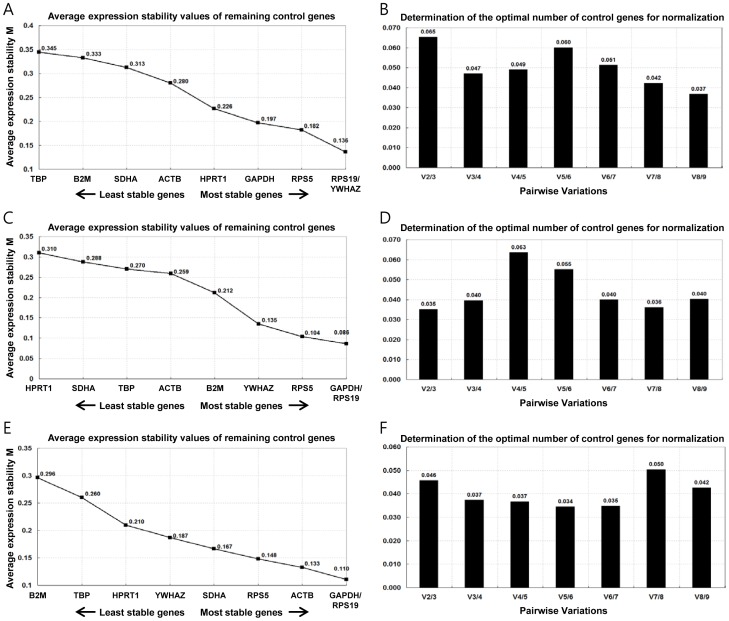
GeNorm analysis of the total sample, control, and icv-STZ groups. Average expression stability (M) of 9 candidate reference genes and the best combination of 2 genes were calculated (A, C, and D). Lower M values indicate more stable expression. Determination of the optimal number of reference genes for normalization was conducted (B, D, and F). The geNorm program calculated the normalization factor (NF) from at least 2 genes and the variable V defines the pair-wise variation between the 2 sequential NF.

The expression stability of the 9 candidate reference genes was analyzed by the geNorm program. GeNorm selects the most suitable reference genes by calculating the stability values (M values) of the tested samples. The M value is calculated by the average pair-wise variation of a particular gene compared with all other control genes. Thereafter, the gene with the highest M value is excluded, and then new M values are calculated from the remaining genes. The genes with high M values are highly variable and less stably expressed, and the genes with low M values possess low variability and are stably expressed. The average M values of the 9 reference genes in the 3 tested sample groups are displayed in Fig. 1. The reference genes *RPS19* and *YWHAZ* were identified as the 2 most stably expressed genes in the total sample group (Fig. 1A); *GAPDH* and *RPS19* were identified in the control group and icv-STZ group (Fig. 1C and 1E).

The geNorm program was also utilized to calculate the optimal number of required reference genes to obtain reliable results from RT-qPCR studies. This calculation was performed by analysis of the pair-wise variation (V value) of sequential normalization factors (NF) with an increasing number of reference genes (NFn and NFn+1) in all tested sample groups (Fig. 1B, 1D, and 1F). The original paper using the geNorm program proposed 0.15 as the cut-off value; implying that if a value below this is obtained an additional reference gene is not required [1]. The pair-wise variation V2/3 was lower than 0.15 in all sample groups; therefore, additional reference genes were not required to calculate the NF.

#### b) NormFinder analysis

NormFinder is a tool to identify the optimal stable reference genes using a model-based approach. NormFinder calculated the stability value and standard error according to the expression variation of the candidate reference genes; stably expressed genes, which have less varied expression levels, have lower stability values [9]. Analysis of our data showed that *RPL19* (0.090) was the most stable reference gene with the lowest stability value, whereas *YWHAZ* (0.148), *ACTB* (0.148), *RPS5* (0.153), *GAPDH* (0.188), *SDHA* (0.196), *HPRT1* (0.205), *B2M* (0.208), and *TBP* (0.221) had respectively increasing stability values in the total group (Table 1). *GAPDH* (0.051) and *ACTB* (0.022) were the most stable reference genes in the control group and the icv-STZ group, respectively.

**Table 1:** Gene stability value calculations by NormFinder.

Total sample group		Control group		Icv-STZ group	
Gene name	Stability value	Gene name	Stability value	Gene name	Stability value
*RPS19*	0.090	*GAPDH*	0.051	*ACTB*	0.022
*YWHAZ*	0.148	*RPS5*	0.112	*GAPDH*	0.073
*ACTB*	0.148	*RPS19*	0.119	*RPS19*	0.077
*RPS5*	0.153	*B2M*	0.153	*SDHA*	0.083
*GAPDH*	0.188	*YWHAZ*	0.155	*RPS5*	0.094
*SDHA*	0.196	*ACTB*	0.186	*YWHAZ*	0.161
*HPRT1*	0.205	*TBP*	0.191	*HPRT1*	0.193
*B2M*	0.208	*SDHA*	0.197	*B2M*	0.260
*TBP*	0.221	*HPRT1*	0.244	*TBP*	0.261

#### c) BestKeeper analysis

The BestKeeper program is also an Excel-based software tool like geNorm and NormFinder. The program determines the coefficient of correlation analysis for all pairs of candidate reference genes (≤10 genes), and calculates the % coefficient of variation (CV) and standard deviation (SD) for each candidate gene’s crossing point (CP) value (the raw quantification cycle value; Cq) [10]. Based on these indices, the most stable reference gene for the accurate normalization of the RT-qPCR data was determined (Table 2). The *TBP* gene had the lowest CV (1.00) and SD (0.27) values of the candidate reference genes; indicating that it was stably expressed across all tested samples. However, *TBP* had a very low coefficient of correlation value (0.836) in the total sample group; indicating that its expression did not correlate well with the expression patterns of the other candidate reference genes. On the contrary, *RPS5* had a high coefficient of correlation value (0.981) and high CV (3.05) and SD (0.61) values. Therefore, *YWHAZ*, *RPS19*, *HPRT1*, and *GAPDH* were stably expressed with high coefficient of correlation, and low CV and SD values in the total sample group. Similarly, *GAPDH*, *HPRT1*, *RPS5*, and *RPS19* in the control group and *GADPH*, *ACTB*, *YWHAZ*, and *SDHA* in the icv-STZ group were stable reference genes.

**Table 2:** Expression stability analysis of the reference genes by BestKeeper.

	Total sample group			Control group			Icv-STZ group		
Genes	R	CV	SD	R	CV	SD	R	CV	SD
*GAPDH*	0.939	2.96	0.55	0.975	1.28	0.23	0.986	3.02	0.58
*ACTB*	0.916	1.85	0.36	0.533	0.80	0.15	0.994	2.82	0.54
*HPRT1*	0.935	2.63	0.60	0.969	1.93	0.43	0.957	3.04	0.69
*RPS5*	0.981	3.05	0.61	0.979	1.59	0.31	0.996	3.46	0.70
*RPS19*	0.983	2.80	0.53	0.930	1.44	0.27	0.995	3.42	0.66
*TBP*	0.836	1.00	0.27	0.496	0.58	0.15	0.879	1.50	0.40
*YWHAZ*	0.952	2.46	0.53	0.828	1.16	0.25	0.970	2.98	0.65
*B2M*	0.857	1.67	0.39	0.687	0.70	0.16	0.889	2.56	0.60
*SDHA*	0.859	1.70	0.39	0.651	1.23	0.28	0.987	2.17	0.46

Finally, we selected the most stable reference genes in the total sample group (*RPS19* and *YWHAZ*), control group (*GAPDH* and *RPS19*) and icv-STZ group (*ACTB* and *GAPDH*) by using the combined data from the geNorm, NormFinder, and BestKeeper programs.

### Comparative Analysis of most and Least Stable Reference Genes on the Normalization of *APP* and *MAPT* Genes

**Figure 2:**
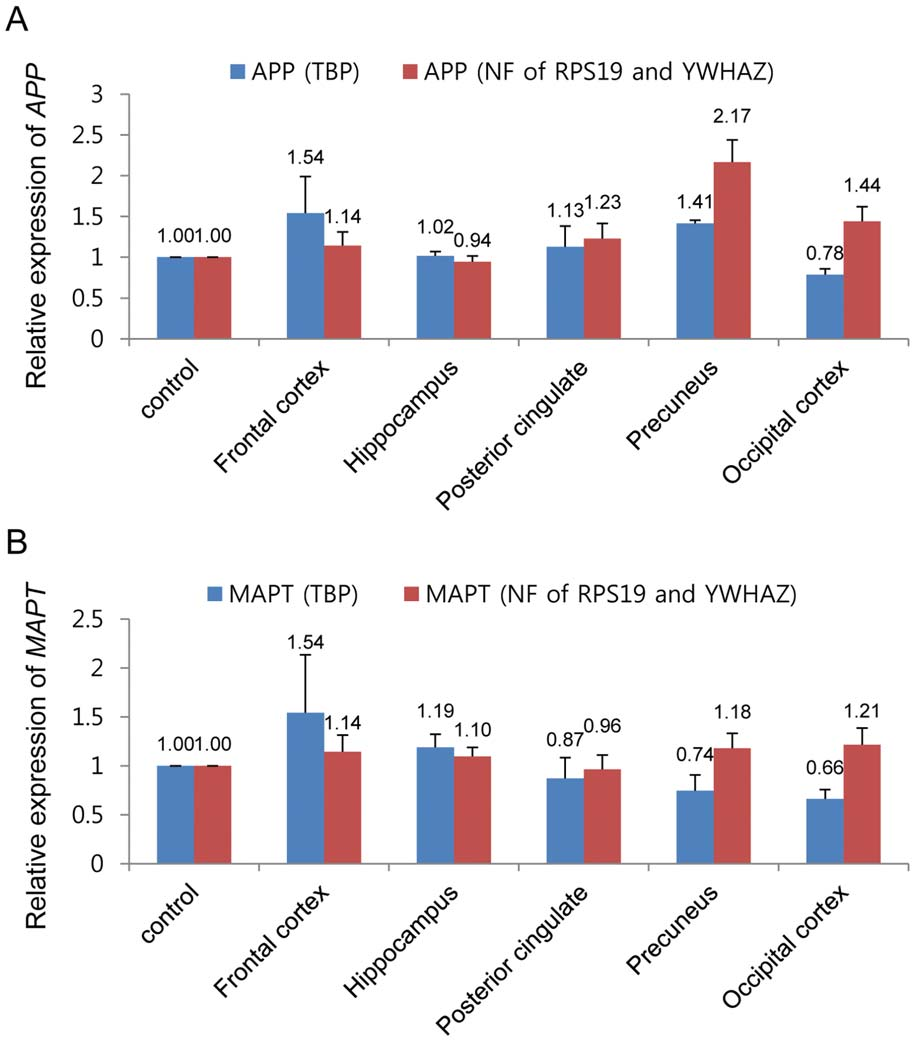
Relative expression of *APP* and *MAPT* in total sample group. The expression level of *APP* (A) and *MAPT* (B) were normalized by normalization factor (NF) of selected most stable reference genes (red) and least stable gene (blue). NF was calculated as the geometric mean of expression level of the *RPS19* and *YWHAZ*. The least stable reference gene (TBP) was selected by geNorm and NormFinder programs.

To demonstrate the importance of using carefully selected normalization genes to estimate the relative expression of the *APP* and *MAPT* genes, we tested the 2 most stable genes (NF of *RPS19* and *YWHAZ*) and least stable gene (*TBP*) for normalization in the total sample group (Fig. 2A and 2B). The relative expression of the *APP* gene following normalization by NF of the *RPS19* and *YWHAZ* genes showed increased levels across the icv-STZ group compared with the control group, except in the hippocampal tissue (Fig. 2A). In particular, the expression level was more than double in the icv-STZ precuneus sample. However, there was no significant difference in the expression level of the control group and the icv-STZ group in the frontal cortex, hippocampus, and posterior cingulate. When the *TBP* gene was used for normalization, the *APP* gene showed increased expression levels in the frontal cortex, posterior cingulate, and precuneus, whereas decreased expression levels were observed in the occipital cortex. These normalization results reveal significant difference in the frontal cortex, precuneus, and occipital cortex. Furthermore, the expression level of the *APP* gene showed the opposite pattern in the hippocampus and occipital cortex. The *MAPT* gene demonstrated increased expression patterns in the frontal cortex, hippocampus, precuneus, and occipital cortex; and decreased expression patterns in the posterior cingulate compared to the normalization against the NF of *RPS19* and *YWHAZ*. However, the expression levels of the *MAPT* gene were not significantly different between the control group and the icv-STZ group. Following normalization using the *TBP* gene, the *MAPT* gene showed increased expression patterns in the frontal cortex and hippocampus, and decreased expression patterns in the posterior cingulate, precuneus, and occipital cortex. Among these, the expression of the *MAPT* gene showed opposing results in the precuneus and occipital cortex.

## Discussion

In recent studies, the RT-qPCR method has been widely used for gene expression analysis because of its specificity, sensitivity, and accuracy. To obtain accurate gene expression data from RT-qPCR experiments, stably expressed reference genes are needed to normalize the target gene’s transcript levels. Therefore, studies of suitable reference genes are essential for accurate normalization and data analysis. Indeed, reference genes have been widely analyzed in various species (including animals and plants), tissues, and under different experimental conditions; and these reference genes have been widely applied for the accurate quantification of target gene expression levels.

The rhesus monkey (*Macaca mulatta*) and cynomolgus monkey (*Macaca fascicularis*) are widely used animal models for the studying neurodegenerative diseases such as AD, PD, Huntington’s disease, and amyotrophic lateral sclerosis [11]–[15]. However, only a few studies on the selection of suitable reference genes have been performed on the rhesus monkey brain and organs [16]–[18]. Furthermore, to our knowledge, studies on the selection of suitable reference genes have not yet been performed on brain and organ tissues of the cynomolgus monkey. Therefore, more accurate and detailed reference gene information is required for the accurate normalization of expression levels of neurodegenerative disease-related genes in cynomolgus monkey brain tissues.

In this study, we identified the most stable reference genes from 9 commonly used candidate reference genes in the brain tissues of cynomolgus monkey using the geNorm, NormFinder, and BestKeeper programs. And also, to provide a more useful and reliable information for studies of various gene expression in the cynomolgus monkey, we carried out selection of appropriate reference genes in the three groups such as total sample group, control group, and icv-STZ group. Although the rankings of the candidate reference genes in the 3 programs showed slightly different patterns between each sample group, there were similarities between each sample group with respect to the composition of the high ranked genes from each program. This difference in the stability ranking of the candidate reference genes may be due to the different algorithms and analytical procedures utilized by the 3 programs. Therefore, the most suitable reference genes for the accurate normalization of target gene expression were selected by combining the data obtained for the top 3 reference genes from each program in each sample group. With respect to the total sample group, *RPS19*, *YWHAZ*, and *RPS5* were the top 3 genes from the geNorm program, *RPS19*, *YWHAZ*, and *ACTB* from the NormFinder program, and *YWHAZ*, *RPS19*, *HPRT1*, and *GAPDH* from the BestKeeper program. Among these genes, *RPS19* and *YWHAZ* were present in all the programs. These 2 genes were ranked in the top 2 in the geNorm and BestKeeper programs; comparatively, *HPRT1* and *GAPDH*, showed low stability values in the geNorm and NormFinder programs, and *ACTB* showed low stability values in the geNorm program. Therefore, *RPS19* and *YWHAZ* were selected as the most suitable reference genes in the total sample group. Similarly, we selected *GAPDH* and *RPS19* in the control group, and ACTB and GAPDH in the icv-STZ group. Previous studies have indicated that commonly used reference genes such as *ACTB*, *GAPDH*, and *B2M* are not stably expressed across tissue types and experimental conditions [4], [19], [20] . However, our results showed that *ACTB* and *GAPDH* were the most suitable reference genes in the control group and the icv-STZ group for the cynomolgus monkey. The different results regarding the reference genes may be due to different of species, tissue types, and experimental conditions. Therefore, the selection of appropriate reference genes must be performed before analyzing the target genes. Specifically, it is important to identify appropriate reference genes for the accurate normalization of target genes in a variety of tissues and experimental conditions in the cynomolgus monkey, to ensure this animal model is a suitable organism for studies of human neurodegenerative diseases.

In this study, the expression levels of *APP* and *MAPT* were measured by normalization with the NF of *RPS19* and *YWHAZ* in the total sample group (Fig. 2A and 2B). The expression levels of the *APP* and *MAPT* genes in the control and icv-STZ samples did not differ in most samples; however, the expression levels of the *APP* gene did differ in the precuneus and occipital cortex tissues. Previous studies have revealed significantly reduced expression levels of the *MAPT* gene and significantly increased expression levels of the *APP* gene in frontal cortex, hippocampus, and hypothalamus samples of human AD relative to the control [21], [22]. These altered expression patterns may be explained by spontaneous recovery of the cynomolgus monkey against brain damage induced by STZ injection because in our previous study, the icv-STZ cynomolgus monkey had a recovered voxel count of brain parenchyma (gray matter+white matter) [8].

Finally, to evaluate the suitability of the selected reference genes in this study, we performed comparative analysis of the expression levels of the *APP* and *MAPT* genes by normalization using the least stable reference gene (*TBP*; Fig. 2A and 2B). These results indicated that normalization using inappropriate reference genes could lead to misinterpretation of target gene expression data. Indeed, diametrically opposed expression patterns of *APP* were observed in the hippocampus and occipital cortex and of *MAPT* in the precuneus and occipital cortex. Moreover, recent studies also demonstrated that target gene expression data following normalization using unstable reference genes showed significantly different results compared with expression data following normalization with suitable reference genes [2], [23], [24]. Unfortunately, many studies still use traditional reference genes such as *GAPDH* and *ACTB* or randomly selected single reference genes for the normalization of gene expression, which is likely to jeopardize the accuracy of the analyzed data [25]–[29]. Therefore, studies aimed at the selection of appropriate reference genes are essential to ensure the accuracy of quantification data of target gene expression obtained using RT-qPCR experiments.

This study was the first to evaluate suitable reference genes in control and icv-STZ brain tissue of cynomolgus monkeys using the geNorm, NormFinder, and Bestkeeper programs. Our results showed that the *RPS19* and *YWHAZ* genes in the total sample group, *GAPDH* and *RPS19* in the control group, and *ACTB* and *GAPDH* in the icv-STZ group were the most appropriate reference genes. Moreover, we validated the normalization accuracy of the most stable reference genes (*RPS19* and *YWHAZ*) in the total sample group by quantifying the mRNA levels of *APP* and *MAPT*. These results provide reliable information for future quantitative analyses of gene expression, particularly related to AD, in the brain tissue of cynomolgus monkeys.

## Materials and Methods

### Experimental Animals and Sampling

Four healthy 3-year-old, 3∼4 kg female cynomolgus monkeys were used. Their origin is Vietnam. All animals were provided by the National Primate Research Center (NPRC) of Korea. This monkey was kept in an indoor individual cage and fed commercial monkey chow (Harlan) supplemented daily with various fruits, and supplied water ad libitum. Environmental conditions were controlled to provide a temperature of 24±2°C, a relative humidity of 50±5%, 100% fresh air at a rate of ≥12 room changes per hour, and a 12∶12 h light:dark cycle. Their health was monitored by the attending veterinarian consistent with the recommendations of the Weatherall Report.

Four cynomolgus monkeys were divided into 2 groups, namely, the icv-STZ group (n  = 2) and the control group (n  = 2). In the icv-STZ group, the STZ was injected into the cerebrospinal fluid (CSF) via the cerebellomedullary cistern (CM) using a 25-g spinal needle on day 1, 7, and 14. The monkeys were treated with 2 mg/kg STZ dissolved in 0.3 mL of normal saline. The control monkeys were injected with the same volume of normal saline. The animals were sacrificed at 20 weeks after the STZ or saline treatments following deep anesthesia using ketamine (20 mg/kg) by intramuscular injection, and perfusion with diethylpyrocarbonate (DEPC)-treated cold phosphate buffered saline (PBS) was conducted via the common carotid artery with RNase inhibitors to inhibit blood contamination and promote recovery of intact RNA molecules from the tissue samples.

### Ethics Statement

All procedures and use of monkeys were approved by the Korea Research Institute of Bioscience and Biotechnology (KRIBB) Institutional Animal Care and Use Committee (Approval No. KRIBB-AEC-11010).

### Total RNA Isolation and cDNA Preparation

Total RNA was extracted from the 2 matched-pair brain samples (5 regions) of control cynomolgus monkeys and those who had received intracerebroventricular injection of streptozotocin (icv-STZ) (total 20 samples) using the RNeasy Mini kit (Qiagen) according to the manufacturer’s instructions. The RNase-free DNase set (Qiagen) was used to eradicate DNA contamination from the total RNA preparations. Total RNA was quantified using a NanoDrop® ND-1000 UV-Vis Spectrophotometer. Moloney-Murine Leukemia Virus (M-MLV) reverse transcriptase was used for the reverse transcription reaction in the presence of the RNase inhibitor (Promega), with an annealing temperature of 42°C. We performed PCR amplification without the reverse transcription reaction using pure RNA samples (no RT control), and determined that the prepared mRNA samples did not contain genomic DNA.

### Primer Design and Standard Curve Analysis

For the development of specific primers for the 9 candidate reference genes and 2 target genes (*APP* and *MAPT*), primer pairs were designed using the Primer3 program (http://frodo.wi.mit.edu/primer3/; Table 3) [30]. The gene sequences were obtained from our previous study sequencing the transcriptome of the cynomolgus monkey [31]. BLAST searches were performed to confirm the gene specificity of the primer sequences, and the results showed the absence of multi-locus matching at individual primer sites. Most primers spanned at least 2 exons or have a great size of introns sequence between forward and reverse primer in order to avoid false-positive amplification of contaminating genomic DNA in the RNA samples. The nucleotide sequences of the RT-PCR products for the 9 reference genes and 2 target genes were obtained by using standard cloning and sequencing procedures (Fig. S1). Amplification efficiencies and correlation coefficients (R^2^ values) of the 11 genes were generated using the slopes of the standard curves obtained by serial dilutions. Standard curves with a 10-fold dilution series were used to calculate the amplification efficiency (Table 3). The amplification efficiency was calculated according to the formula: efficiency (%) = (10^(−1/slope)^−1)×100. The efficiency range of the real-time RT-PCR amplifications for all the tested genes was 88%–100%.

**Table 3:** Primers for the 11 genes and parameters derived from RT-qPCR data analysis.

Abbreviation	Gene name	Primer*Forward(F)/Reverse(R)	Exon(s)	Ampliconsize (bp)	PCRefficiency(%)	R^2^	NTC**(Cq)
*ACTB*	*Beta-actin*	F: ACAGAGCCTCGCCTTTGCR: CACGATGGAGGGGAAGAC	1^st^2^nd^	160	92	0.99094	32
*B2M*	*Beta-2-microglobulin*	F: GTCTCGCTCAGTGGCCTTAR: GTGGATGGCGTGAGTAAACC	1^st^2^nd^	101	88	0.99174	32.5
*GAPDH*	*Glyceraldehyde-3-phospate dehydrogenase*	F: ACAACAGCCTCAAGATCGTCAGR: ACTGTGGT/CATGAGTCCTTCC	6^th^7^th^/8^th^	112	90	0.99273	34.18
*HPRT1*	*Hypoxanthine phosphoribosyltransferase 1*	F: GACCAGTCAACAGGGGACAR: AAAGTCTGCATCGTTTTGCC	4^th^6^th^	116	92	0.99380	32.6
*RPS5*	*Ribosomal protein S5*	F: GTCCTGGTGAACGCCATCR: TCAGCAATGGTCTTAATGTTCC	4^th^5^th^	182	100	0.99338	33.5
*RPS19*	*Ribosomal protein S19*	F: AGCTTGCTCCCTACGATGAGR: GACGAGCCACACTCTTGGA	3^rd^4^th^	174	93	0.99581	36.04
*SDHA*	*Succinate dehydrogenase complex subunit A*	F: AAACCAAATGCTGGAGAAGAATR: TCCGTAGAGCTTGCTGATTT	11^th^12^th^	180	88	0.99386	N.d.
*TPB*	*TATA box binding protein*	F: CCACTCCACTGTATCCCTCCR: TATATTCAGCATTTCGGGCA	3^rd^4^th^	174	97	0.99045	35.51
*YWHAZ*	*Tyrosine 3-monooxygenase/tryptophan 5-monooxygenase activation protein, zeta polypeptide*	F: AGCAGATGGCTCGAGAATACAR: GTCATCACCAGCGGCAAC	2^nd^ 3^rd^	185	97	0.99120	38.44
*APP*	*Amyloid beta (A4) precursor protein*	F: TACCGCTGCTTAG/TTGGTGAR: CTCACTGCACGT/CTCTTTGG	3^rd^/4^th^4^th^/5^th^	138	90	0.99179	32.3
*MAPT*	*Microtubule-associated protein tau*	F: TAGCAACGTCCAGTCCAAGTR: TCTGTCCTTGAAGTCCAGCT	11^th^13^th^	196	90	0.99080	N.d.
					

* If a primer is located on 2 exons, the junctions are shown with a virgule.

** No template control.

N.d: Not detected.

### RT-qPCR Amplification

RT-qPCR using SYBR Green was performed using a Rotor Gene Q thermocycler (Qiagen). In each run, 1 µL of cDNA was used as a template for each reaction. The samples were added to 19 µL of the reaction mixture, containing 7 µL H_2_O, 10 µL RotorGene SYBR Green PCR mastermix (Qiagen), and 1 µL each of the forward and reverse primers. RT-qPCR amplification of the 11 genes was performed for 40 cycles of 94°C for 5 sec and 60°C for 10 sec. The amplification specificity of each RT-qPCR assay was confirmed by melting curve analysis. The temperature range for analysis of the melting curves was 55°C to 99°C for 5 sec. As shown in Fig. S2, each primer pair showed a single, sharp peak, thereby indicating that the primers amplified only one specific PCR product. The no-template control (NTC) was detected with any of the tested genes except for SDHA and MAPT. However, the NTC of these 9 genes was amplified over the 32 cycles (Table 3). Therefore, it did not affect the amplification of the target gene. These data generated 3 independent experiments.

### Characterization of the Analysis Programs

The geNorm program [1] provides a measure of gene expression stability (M value), which is the mean pair-wise variation between an individual gene and all other tested control genes. This method differs from model-based approaches, as it compares genes based on the similarity of their expression profiles. Cq values were converted to scale expression quantities using the ΔCq method and recorded in the geNorm program, which then ranked the genes based on their M values; the gene with the most stable expression has the lowest value. NormFinder [9] focuses on finding the 2 genes with the least intra- and inter-group expression variation or most stable reference gene in the intra-group expression variation. A BestKeeper [10] index was created using the geometric mean of each candidate gene’s Cq values. This index was then compared to each individual candidate reference gene by pair-wise correlation analyses, with each combination assigned a value for the Pearson correlation coefficient (r), probability (p), SD, and CV.

### MIQE Guidelines

All the experiments were performed according to the Minimum Information for Publication of Quantitative Real-Time PCR Experiments (MIQE) guidelines [32].

## Supporting Information

Figure S1 Nucleotide sequences of the candidate reference genes and target genes from the cynomolgus monkey brain.
(TIF)

Figure S2 Melting curve analyses of the candidate reference genes and target genes in the total samples.
(TIF)
