# Supplementary figures and images for: Selection of Appropriate Reference Genes for RT-qPCR Analysis in a Streptozotocin-Induced Alzheimer’s Disease Model of Cynomolgus Monkeys (Macaca fascicularis)
Source: PLoS One. 2013 Feb 14;8(2):e56034. doi: 10.1371/journal.pone.0056034 (PMC3573079; doi:10.1371/journal.pone.0056034)

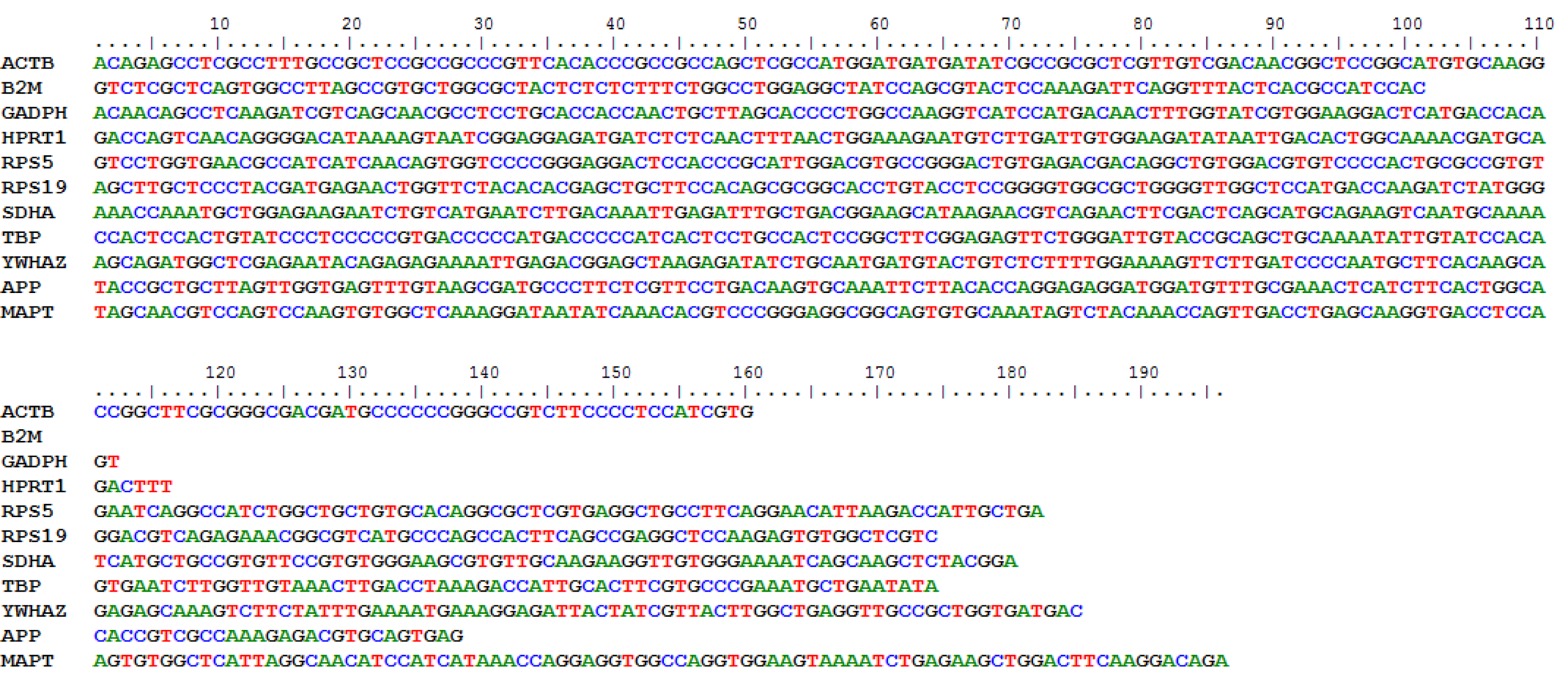

Supplement: Figure S1 — Nucleotide sequences of the candidate reference genes and target genes from the cynomolgus monkey brain. (TIF) [file pone.0056034.s001.tif]

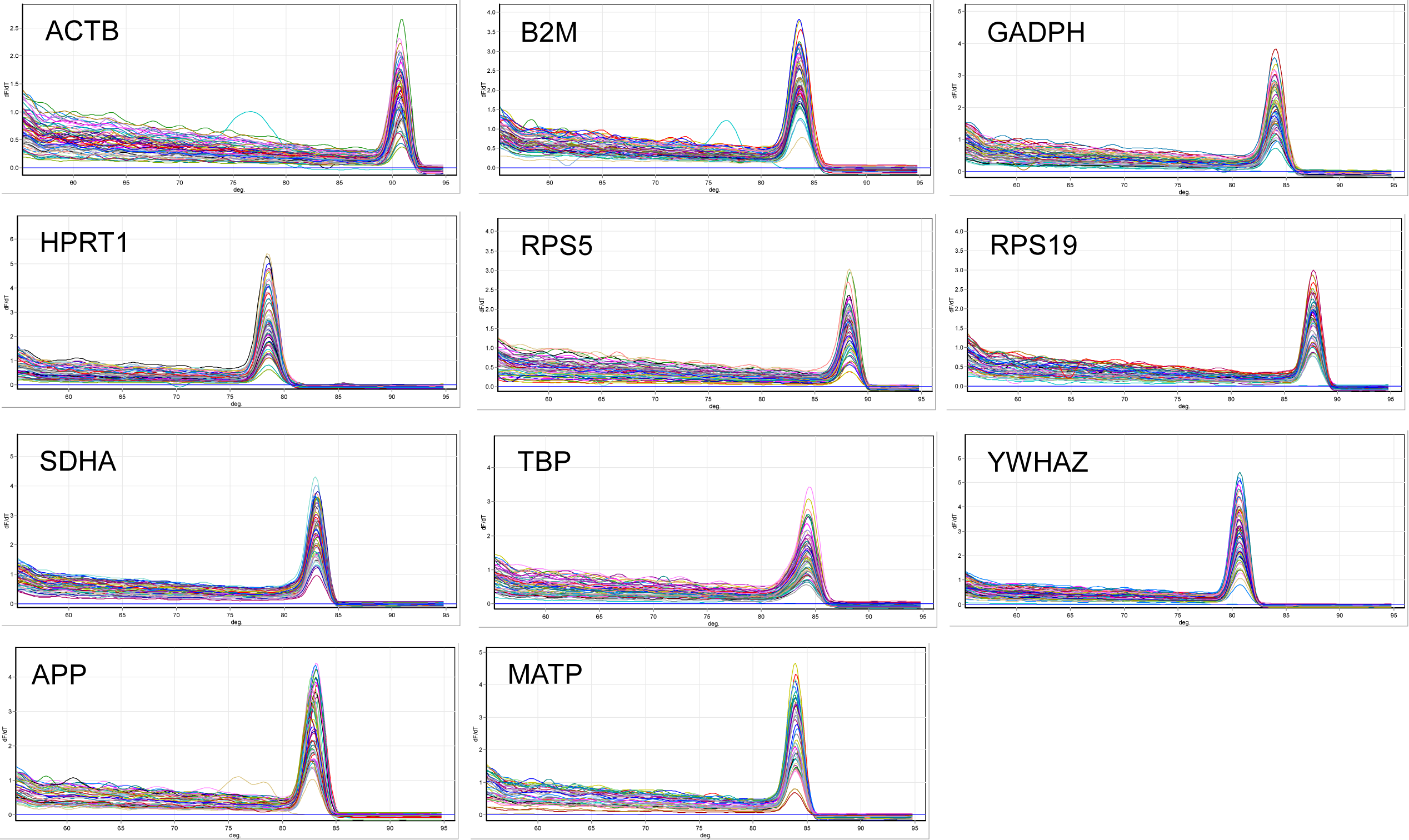

Supplement: Figure S2 — Melting curve analyses of the candidate reference genes and target genes in the total samples. (TIF) [file pone.0056034.s002.tif]
